# Medicine shortages: Product life cycle phases and characteristics of medicines in short supply—A register study

**DOI:** 10.3389/fphar.2022.943249

**Published:** 2022-06-27

**Authors:** Kati Sarnola, Heini Kari, Hanna Koskinen

**Affiliations:** Research at Kela, Social Insurance Institution of Finland, Helsinki, Finland

**Keywords:** medicine shortage, product life cycle, age, reimbursement, register study

## Abstract

**Introduction:** Product life cycle refers to all phases of a product from development to active market phase and finally the phase in which products possibly exit the market. The product life cycle of medicines in short supply has not been studied in depth, although there is some indication of mature products and products with lower prices and profit margins being exposed to shortages more often. The aim of this study was to examine the product life cycle phases and characteristics of medicines in short supply as well as the features of medicine shortages in Finland from 2017 to 2019.

**Material and methods:** Register data on medicine shortages of human medicinal products from 2017 to 2019 was combined with timely data on marketing authorizations and reimbursement status to gain data on product life cycle phases and characteristics (e.g., the age and the reimbursement status) of medicines in short supply and the features of medicine shortages. The data were analyzed in descriptive manner using appropriate statistical testing.

**Results:** 3,526 shortages were reported during the 3-year study period and the number of shortages increased annually. The average duration of a shortage was 83 days and shortages affected 660 active pharmaceutical ingredients. Most often, shortages occurred with medicines affecting the nervous system, the cardiovascular system, and the genitourinary system. A majority of shortages (*n* = 2,689) was reported in the reimbursable medicines group, where shortages increased as the number of patients receiving reimbursements increased (*p* < 0.001). In the reimbursable medicines group, shortages most commonly involved medicines aged 15–19, 20–24, and 25–29, whereas with both reimbursable and non-reimbursable products the shortages most often occurred in medicines aged 50–54. The frequency of shortages differed between the groups (*p* < 0.001) when both age and reimbursement status were taken into account.

**Conclusion:** Medicine shortages are common and affect commonly used medicines. Product life cycle phase has an effect on the frequency of shortages: Reimbursable medicines and medicines exposed to changes in life cycle are more likely to face a shortage. The impacts of product life cycle on the availability of medicines and medicine shortages should be studied in more detail.

## Introduction

The first medicine shortage, a shortage of insulin, was reported a century ago. Today, medicine shortages pose an increasing challenge to patient care worldwide (U.S. Food and Drug Administration 2019; Shukar et al., 2021). The causes of shortages have been studied and mitigation strategies have been developed (U.S. Food and Drug Administration 2019; Shukar et al., 2021) because medicine shortages have major impacts on patient care, the workload of health-care professionals, and even on pharmaceutical costs (Dill and Ahn 2014; Mazer-Amirshani et al., 2014; Fox and Tyler 2017; Phuong et al., 2019).

The causes of medicine shortages often remain unclear, partly due to the complexity of the issue and the plurality of the causes (Shukar et al., 2021). Earlier research has recognized supply and demand issues and regulatory issues as major determinants of shortages (Dill and Ahn 2014; Alsheikh et al., 2016; Fox and Tyler 2017; Heiskanen et al., 2017; Phuong et al., 2019; Shukar et al., 2021). Furthermore, reports and studies indicate that products with lower prices and profit margins and mature products that have lost their exclusive selling rights might be more exposed to shortages (Fox and Tyler 2017; Dave et al., 2018; U.S. Food and Drug Administration 2019; Tapanila et al., 2021), as the manufacturers’ motivation to keep products in the market may be lower with less profitable medicines. A retrospective cohort study has reported the lowest priced medicines being at a substantially elevated risk of shortage (Dave et al., 2018). In part, mature products may be more exposed to shortages because the market does not reward manufacturers for investing in quality and back-up systems when it comes to mature products (U.S. Food and Drug Administration 2019). Minimizing investments might eventually lead to quality issues that potentially cause shortages. Previous findings by the United States Food and Drug Administration (FDA) define mature products as those with the median time of 35 years since first approval (U.S. Food and Drug Administration 2019). However, a uniform definition of mature products is lacking, resulting in a lack of comparative information between countries and markets.

Product life cycle refers to all development, regulatory and optimization procedures during the lifespan of a medicine (Bauer and Fischer 2000; Langedijk et al., 2016; Stevens et al., 2020; Bere 2022). Life cycle includes the pre-submission phase (including development to non-clinical and clinical testing), the evaluation phase (including marketing authorization, and price and reimbursement evaluations, negotiations, and implementation), the post-marketing authorization phase (including post-marketing authorization surveillance, and optimization of life cycle, and patent protection) and finally the market exit phase. Critical aspects in terms of availability of medicines and medicine shortages exist in each phase. However, the impacts of product life cycle on the availability of medicines and medicine shortages have not been studied in depth or published in scientific journals.

The aim of this study was to examine the product life cycle phases and characteristics of medicines in short supply as well as the features of medicine shortages in Finland from 2017 to 2019. More specifically, we studied the frequency and duration of medicine shortages, affected active ingredients and medicine groups, and if and how age and reimbursement status of the products in short supply affect the frequency of shortages. The period 2017–2019 was selected to cover the most recent years prior to the COVID-19 pandemic.

## Material and methods

### Context and data

The data were formed from registers held by two national authorities: Finnish Medicines Agency (Fimea) and the Social Insurance Institution of Finland (Kela). Fimea is responsible for, e.g., handling marketing authorizations, the supervision of product life cycle from classification to marketing promotion, and for collecting and reporting data on medicine shortage notifications (Finnish Medicines Agency 2022a). Kela is responsible for, e.g., social security coverage (including medicine reimbursements) of all residents regardless of age, wealth or address (Kruuti, 2021; The Social Insurance Institution of Finland 2022a). In Finland, the National Health Insurance scheme covers some of the costs of necessary prescription medicines and some over-the-counter products and basic ointments prescribed by a physician (Kruuti, 2021). Generic substitution and reference price system are in use. The reimbursement system is vital in steering rational prescribing and use of pharmacotherapies and in moderating pharmaceutical costs (Närhi and Asola 2021).

In this study, a medicine shortage accounts for a shortage notification of a human medicinal product from marketing authorization holder to the national authority, Finnish Medicines Agency (Finnish Medicines Agency 2022b). In Finland, shortage reporting is mandatory, on pain of a fine. Multiple notifications can be done for each product. In this study, the data on shortage notifications included the Anatomic Therapeutic Chemical (ATC) classification (World Health Organization 2022) of the product in short supply, the number of shortage notifications from 2017 to 2019, and the start and end dates of each shortage. For simplicity, all shortages that were active on 1 January 2017, were included in the data, even though some shortages may have emerged earlier. Correspondingly, all shortages that were active on 31 December 2019, were included, even though some shortages may have lasted beyond the study period. Shortage notifications with unclear or incomplete ATC codes or missing start and end dates were excluded from the data. Shortage notifications did not contain information on the product name, strength, or package size.

The shortage notifications data were combined with data from Medicinal Products Database (The Social Insurance Institution of Finland 2022b). The database contains information on whether there are reimbursable products within each seven-digit ATC code (e.g., A01AA01). In this study, the term “reimbursable” is used for a medicine/ATC class in which at least one product is reimbursable, regardless of the reimbursement status of other products in the class. The term “non-reimbursable” is used when none of the products in a class are reimbursable. In the reimbursable ATC classes, the number of patients receiving reimbursements was also included in the data (The Social Insurance Institution of Finland 2022c). The number of patients that received reimbursements annually was divided into four somewhat evenly distributed categories: less than 1,000 patients (90 ATC codes of 382 reimbursable groups), 1,000–9,999 patients (111 ATC codes), 10,000–49,999 patients (103 ATC codes), and 50,000 or more patients (78 ATC codes).

The data were enriched with public data from Fimea’s register on the first marketing authorization date for each seven-digit ATC code in order to calculate the age of the products (Finnish Medicines Agency 2022c). The first marketing authorizations in Finland were granted in 1964 (Palva 2015). Therefore, the classification of the product age in years is 0–4, 5–9, 10–14, 15–19, 20–24, 25–29, 30–34, 35–39, 40–44, 45–49, 50–54, and 55 or older, based on the first marketing authorization date of the originator product of each active ingredient. The data were searched in February 2022, presenting the situation at the time.

### Analysis

The data were analyzed with SPSS Statistics for Windows, Versions 27.0 and 28.0 (SPSS Inc., Chicago, IL, United States) using frequencies and percentages for descriptive analysis. Associations between variables were assessed by Kruskal–Wallis’ and Mann-Whitney’s U- test for independent samples and by Pearson’s χ^2^-test between categorical variables. *p*-value of <0.05 was considered statistically significant.

### Ethical consideration

According to the national ethical instructions (Finnish National Board on Research Integrity 2022), this register study did not require ethical statement or permission.

## Results

### Characteristics of medicines in short supply and medicine shortages

During the 3-year study period, there were 3,539 shortage notifications. Thirteen notifications were excluded from the data due to incorrect or lacking information. The final data consisted of 3,526 medicine shortage notifications. On average, the number equals to more than three shortage notifications a day during the study period. The frequency of shortages increased annually: 817 shortages were reported in 2017, while the number of shortages in 2018 and 2019 were 1,112 and 1,597. Seventeen shortages had started before 2017. The majority of shortages had started in the previous year (2016), but one shortage had reportedly started already in 2015 and another in 2014. Correspondingly, 39 shortages were still active at the end of 2019. The average duration of a reported shortage was 83 days with the median of 60 days.

**FIGURE 1:**
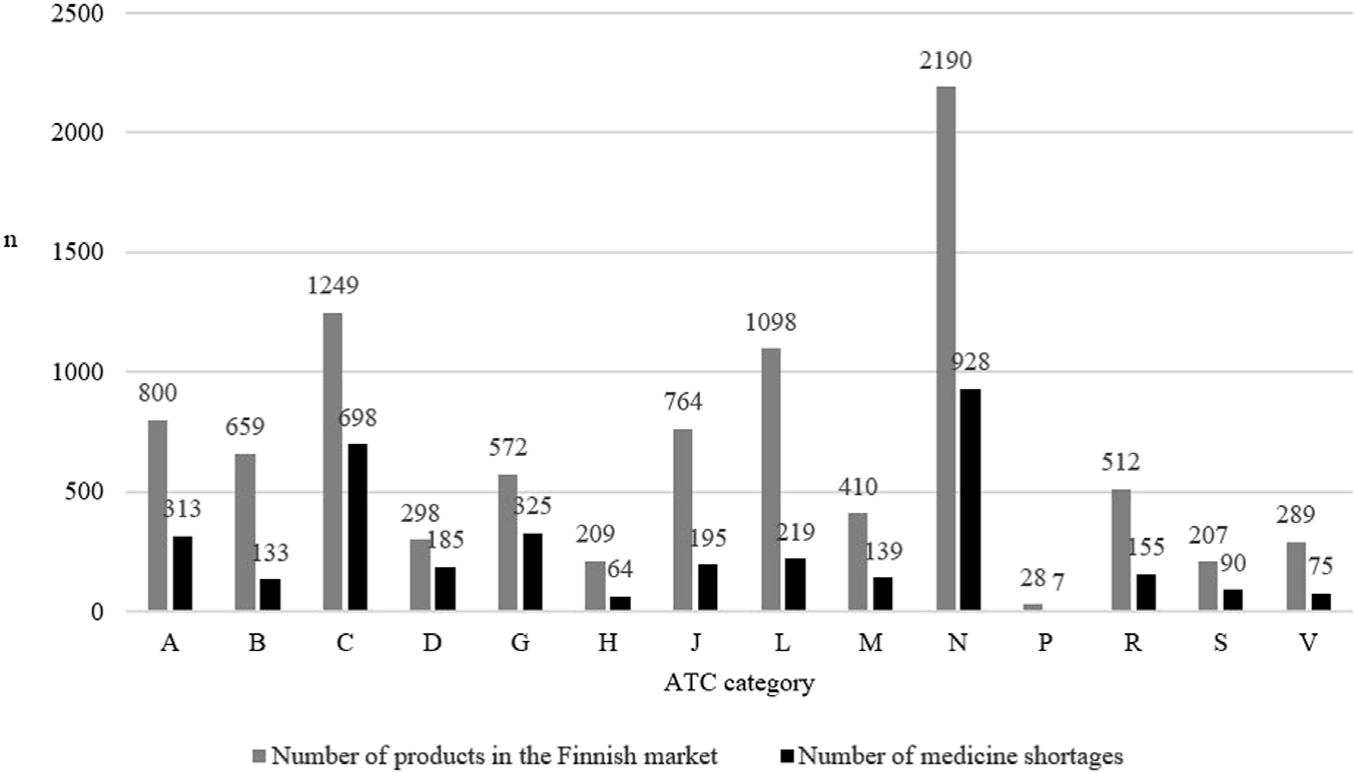
The number of products on the Finnish market in 2022 according to the Anatomic Therapeutic Chemical (ATC) category and the number of medicine shortages in Finland (*N* = 3,526) in each category from 2017 to 2019.

The most common medicine shortages involved medicines affecting the nervous system (ATC code: N, *n* = 928, 26.3% of all shortages), the cardiovascular system (C, *n* = 698, 19.8%), and the genitourinary system (G, *n* = 325, 9.2%) (Figure 1). At the three-digit level of ATC codes, medicine shortages were most common in agents acting on the renin-angiotensin system (C09, *n* = 342, 9.7% of all shortages), analgesics (N02, *n* = 287, 8.1%) and in psychoanaleptics (N06, *n* = 239, 6.8%) (Supplementary Table S1).

Shortages affected 660 active pharmaceutical ingredients. On average, five shortage notifications were made per each API (median being two notifications). Shortages were most often reported of sumatriptan (N02CC01, *n* = 50), rosuvastatin (C10AA07, *n* = 48), candesartan (C09CA06, *n* = 47) and paracetamol (N02BE01, *n* = 47).

### Product life cycle phase of medicines in short supply

**FIGURE 2:**
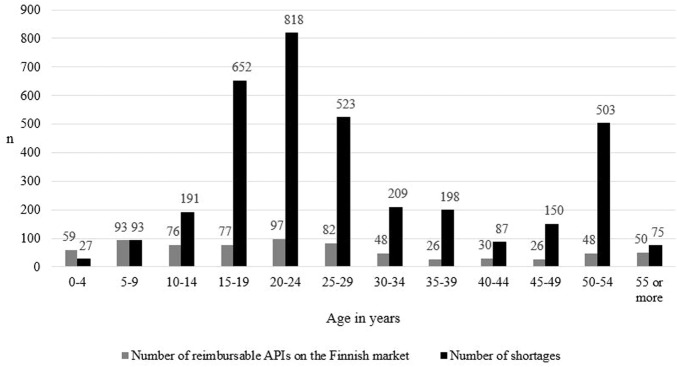
The number of reimbursable active pharmaceutical ingredients (API) marketed in Finland (*N* = 712, based on Kari et at, unpublished), and the number of medicine shortages in Finland from 2017 to 2019 (*N* = 3,526) and the age of the products calculated from the first marketing authorization date. Information on the number of APIs marketed in Finland in classes A10AB03, A10AC03, A11JC, B05AX03, C01BA03, L04AC, N05AL05 and V03AG01 was not available.

Medicine shortages were most common in products aged 20–24 (*n* = 818, 23.2% of all shortages), 15–19 (*n* = 652, 18.5%), and 25–29 (*n* = 523, 14.8%) (Figure 2: the number of medicine shortages according to the age of the products is shown in black bar charts). Notably, shortages were also common in products aged 50–54 (*n* = 503, 14.3%). The difference in the number of medicine shortages in different age groups was statistically significant (*p* < 0.001). In pairwise comparisons, statistically meaningful differences were most often found in comparisons between medicine groups aged 24 or younger, while meaningful differences lacked between pairwise comparisons in medicine groups aged 25 or older (Supplementary Table S2).

Medicines aged 20–24 in short supply (*N* = 818) were most commonly medicines affecting the nervous system (*n* = 225), the cardiovascular system (*n* = 219), and antineoplastic and immunomodulating agents (*n* = 87) (Supplementary Table S3). In medicines aged 15–19 (*N* = 652) and 25–29 (*N* = 523), shortages typically involved medicines affecting the nervous system (*n* = 230 in medicines aged 15–19, and *n* = 140 in medicines aged 25–29), the cardiovascular system (*n* = 107 and *n* = 139), and the genitourinary system (*n* = 71 and *n* = 37). In medicines aged 50–54 (*N* = 503), shortages most often occurred in medicines affecting the nervous system (*n* = 82), alimentary tract and metabolism medicines (*n* = 76), and medicines for cardiovascular diseases (*n* = 56).

A majority of all shortages, 76% in total (*n* = 2,689), were reported in the reimbursable medicines group, while less than one fourth affected the non-reimbursable medicines (*n* = 837) (Supplementary Table S4). In the reimbursable group (*n* = 2,689), shortages were most commonly reported of medicines affecting the nervous system (ATC code N, *n* = 812), the cardiovascular system (C, *n* = 643), the genitourinary system (G, *n* = 219), and alimentary tract and metabolism (A, *n* = 194) and immunomodulating agents (L, *n* = 176). In the non-reimbursable group, shortages most often occurred of medicines affecting the alimentary tract and metabolism (A, *n* = 119), the nervous system (N, *n* = 116), the genitourinary system (G, *n* = 106), in anti-infectives for systemic use (J, *n* = 102), and in dermatologicals (D, *n* = 63).

**FIGURE 3:**
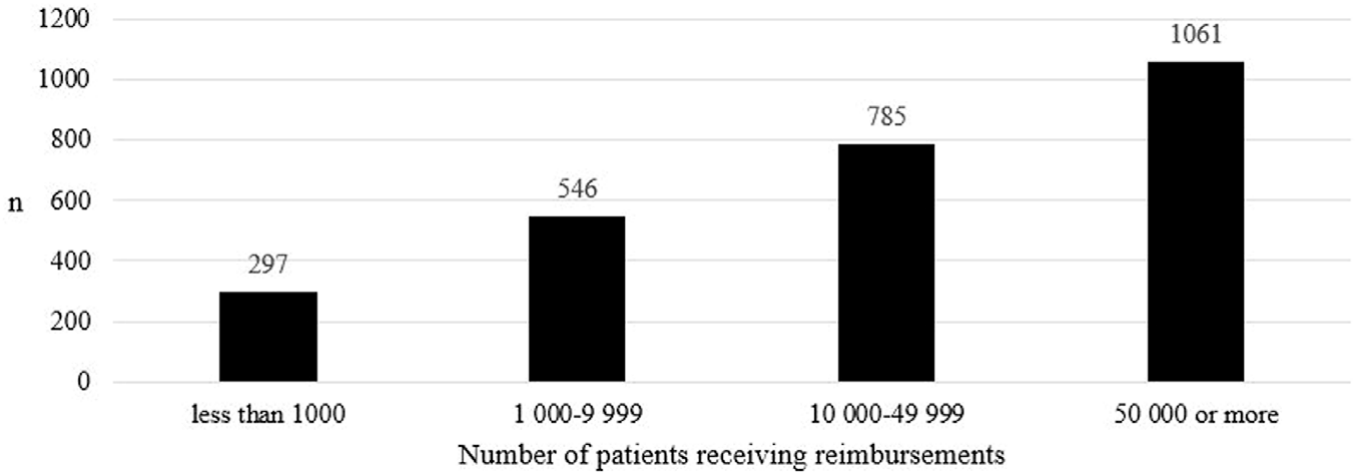
The number of medicine shortages in Finland from 2017 to 2019 in groups including at least one reimbursable product (*N* = 382) according to the number of patients receiving reimbursements in Finland in 2022.

In the reimbursable medicines group, the number of medicine shortages increased as the number of patients receiving reimbursements increased (Figure 3). The difference in the number of medicine shortages between groups was statistically significant in all comparisons between groups (*p* < 0.001).

At the level of active pharmaceutical ingredients, at least one reimbursable product was present in a group in 382 ATC codes of the 660 APIs affected, which accounts for 54% of all reimbursable APIs marketed in Finland (Figure 2: the number of medicine shortages is shown in black bar charts and the number of reimbursable APIs marketed in Finland in grey bar charts). In 278 ATC codes, all products were non-reimbursable.

**FIGURE 4:**
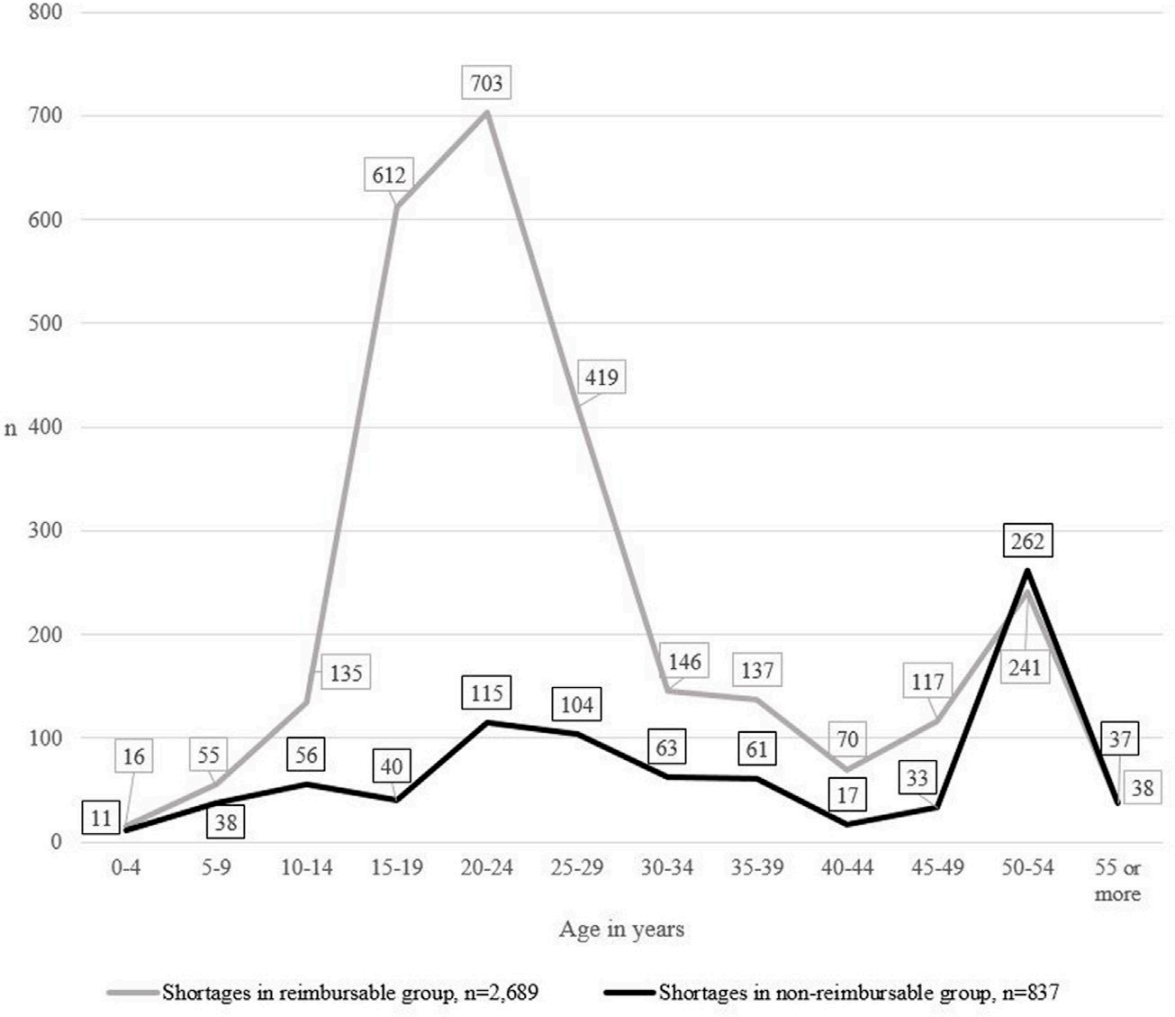
The number of medicine shortages in Finland from 2017 to 2019 (*N* = 3,526), the age of medicines in short supply and the reimbursement status of the group.

When both the age and the reimbursement status of products were taken into account, the frequency of medicine shortages differed between the groups (Figure 4). The difference between groups was statistically significant (*p* < 0.001). In the reimbursable group, shortages peaked in products aged 15–19, 20–24, and 25–29, while shortages in the non-reimbursable group remained stable. In both groups, another, yet smaller, peak was detected in products aged 50 to 54.

## Discussion

According to this study, medicine shortages are common and growing in numbers. Shortages occur of commonly used medicines, such as medicines affecting the nervous system and the cardiovascular system. These results are consistent with earlier studies conducted in Finland (Heiskanen et al., 2015; Tapanila et al., 2021) and other countries (e.g., Pauwels et al., 2014; Dave et al., 2018; Videau et al., 2019; Benhabib et al., 2020; Clark et al., 2020). Even though shortages can often be resolved with alternative, interchangeable products and patients are rarely left without medicines (Heiskanen et al., 2015; Tapanila et al., 2021), they still burden the health care system and health professionals in their daily work, resulting also to possible increase in medicine prices (Fox and Tyler 2017) and undoubtedly an increase to pharmaceutical expenditure (Blankart and Felder 2022). Our results, together with previous results, pinpoint the fact that despite extensive discussion and the implementation of mitigation strategies (e.g., Clark et al., 2020; Musazzi et al., 2020; Shukar et al., 2021), medicine shortages continue to pose a significant threat to pharmaceutical care. There is a growing need to improve the understanding of the determinants of medicine shortages and to find novel strategies to combat the issue of shortages.

To the best of our knowledge, product life cycle phases of medicines in short supply has not previously been studied in depth or published in scientific journals, and our study is the first one to report the differences in the number of shortages of products in different life cycle phases systematically. According to our results, reimbursable medicine groups are more likely to face shortages than non-reimbursable medicine groups. Furthermore, the number of shortages increases as the number of patients receiving reimbursements increases. Our results are in line with previous Finnish studies, indicating that shortages often occur of commonly used medicines (Heiskanen et al., 2015; Tapanila et al., 2021), and with a Canadian study indicating that markets with a high proportion of medicines covered by public insurance programs were more likely to face shortages (Zhang et al., 2020). In addition, our results are in line with the fact that in 2018, 60% of all products on the Finnish market were reimbursable (Ruskoaho 2018), although, in our study, the share of reimbursable medicines group in all shortages was slightly higher, 76%. Notably, according to our results, more than half of reimbursable active pharmaceutical ingredients are affected by shortages, suggesting that critical products may be exposed to shortages as well. In an earlier Finnish study, the number of critical products in shortage was lower (19%), but the study took account both products in the national mandatory reserve supplies and in the WHO Model Lists of Essential Medicines list (Tapanila et al., 2021). Regardless, to draw any further conclusions, additional research on the impacts of price and reimbursement status of products affected by medicine shortages is needed.

Our study is the first one to report statistical differences in the number of shortages of products of different age. According to this study, products aged 15–19, 20–24, 25–29, and 50–54 were most likely to face a shortage. Strikingly, products aged 15–19, 20–24, and 25–29 in short supply mainly belong to the reimbursable medicines group, while products aged 50–54 in short supply include both reimbursable and non-reimbursable products. It seems that in the reimbursable medicines group, shortages peak after the exclusive selling rights and possible additional protection have expired. Typically, this happens approximately 15–20 years after the initial patent was granted (Garattini et al., 2022). Potentially, increased (generic) competition in the market leads to lower profitability and new market positions, due to which some products temporarily exit the market causing a shortage for commercial reasons (Heiskanen et al., 2017; Shukar et al., 2021). According to Canadian studies, a majority of medicines in short supply are manufactured by generic companies (Videau et al., 2019) and markets with a single generic manufacturer are more likely to face a shortage (Zhang et al., 2020). Similarly, a Finnish study reported that medicines in short supply were most often affordable products for which there were one or more generic alternatives available in the market (Tapanila et al., 2021). Our results, together with previous findings, highlight the fact that the product life cycle, the competitive environment and the role and behavior of players in the market, and medicine shortages should be studied in more detail.

According to our study, another peak in shortages is detected in products aged 50–54. Our results are in line with a Finnish and a French study indicating that the medicines is short supply were most often older products (Benhabib et al., 2020; Tapanila et al., 2021). In the United States, reportedly, the median time since first approval of products in short supply in the United States in 35 years (U.S. Food and Drug Administration 2019), also indicating a rise in shortages of older products. In addition, statistically meaningful differences between age groups were most often found in younger medicine groups aged 24 or under. This is logical because changes in the life cycle, such as expiration of exclusive selling rights and changes in reimbursement or competitive environment typically occur within the first 20 years of life cycle. The later phases prior to exiting the market appear to be stable, showing no statistical differences between groups. Our results, together with findings from France and from the United States, indicate that shortages might indicate a permanent exit from the market, as the use and profitability of these products may have been declining over the decades. Overall, our results on the differences in the number of shortages of products of different age support previous findings: Medicine shortages appear to occur simultaneously with changes in the life cycle, for example, when exclusive selling rights expire and in the final phase of life cycle.

The strength of this study is the novel information it provides on the impacts of product life cycle on shortages. Furthermore, the study is based on reliable and comprehensive register data from national authorities. However, this study also has limitations. In this study, we use the term “reimbursable” to refer to classes where at least one product is reimbursable. Unfortunately, the data did not include information on the reimbursement status of each individual product; instead, it only included information on whether at least one product in the class was reimbursable. In addition, the information on reimbursement status and the first marketing authorization dates was searched in February 2022, which means there might have been some changes in comparison to 2017–2019. Nonetheless, we believe that possible changes have been minor and would not have significantly affected the results. It is also noteworthy that the data of this study reflects the situation prior to the COVID-19 pandemic. We acknowledge that distinct results on the occurrence of medicine shortages have also been reported during the pandemic (e.g., American Society of Health System Pharmacists 2020). The pandemic has affected the availability of medicines and, since differences only highlight the diversity of the issue, we believe that research is needed to study the situation prior, during and after the pandemic. Overall, further research on the topic of product life cycle and medicine shortages is also needed to better understand the determinants of shortages and to gain novel mitigation strategies. Although this was a single-country study, product life cycle research in one country produces valuable information on the global impacts as well, since there is little variance between countries in, for example, product age.

## Conclusion

Medicine shortages are common and involve commonly used medicines. Product life cycle phase has an effect on the frequency of shortages, as reimbursable medicines and medicines exposed to changes in life cycle, for example medicines of which exclusive selling rights expire and medicines in the final phase of their life cycle, are more likely to face a shortage. Although this study was conducted in a single country, product life cycle is likely to have similar impacts elsewhere, thus, the impacts of product life cycle on the availability of medicines and medicine shortages should be studied in more detail.

## Data Availability

The raw data supporting the conclusion of this article will be made available by the authors, without undue reservation.

## References

[B1] Alsheikh M. Seoane-Vazquez E. Rittenhouse B. Fox E. R. Fanikos J. (2016). A Comparison of drug shortages in the hospital setting in the united states and saudi arabia: an exploratory analysis. Hosp. Pharm. 51, 370–375. 10.1310/hpj5105-370 27303090 PMC4896345

[B2] American Society of Health System Pharmacists (2020). Drug shortages statistics. Available at: https://www.ashp.org/drug-shortages/shortage-resources/drug-shortages-statistics (Accessed April 14, 2022).

[B3] Bauer H. H. Fischer M. (2000). Product life cycle patterns for pharmaceuticals and their impact on R&D profitability of late mover products. Int. Bus. Rev. 9, 703–725. 10.1016/s0969-5931(00)00028-7

[B4] Benhabib A. Ioughlissen S. Ratignier-Carbonneil C. Maison P. (2020). The french reporting system for drug shortages: description and trends from 2012 to 2018: an observational retrospective study. BMJ Open 10, e034033. 10.1136/bmjopen-2019-034033 PMC7059530 32139487

[B5] Bere N. (2022). How are medicines evaluated at the EMA. Available at: https://www.ema.europa.eu/en/documents/presentation/presentation-how-are-medicines-evaluated-ema-nathalie-bere_en.pdf (Accessed April 13, 2022).

[B6] Blankart K. E. Felder S. (2022). Do medicine shortages reduce access and increase pharmaceutical expenditure? A retrospective analysis of Switzerland 2015-2020. Value Health S1098-3015 (22), 00053–00055. 10.1016/j.jval.2021.12.017 35219600

[B7] Clark S. L. Levasseur-Franklin K. Pajoumand M. Barra M. Armahizer M. Patel D. V. (2020). Collaborative management strategies for drug shortages in neurocritical care. Neurocrit. Care 32, 226–237. 10.1007/s12028-019-00730-7 31077080 PMC7222107

[B8] Dave C. V. Pawar A. Fox E. R. Brill G. Kesselheim A. S. (2018). Predictors of drug shortages and association with generic drug prices: A retrospective cohort study. Value Health 21, 1286–1290. 10.1016/j.jval.2018.04.1826 30442275

[B9] Dill S. Ahn J. (2014). Drug shortages in developed countries--reasons, therapeutic consequences, and handling. Eur. J. Clin. Pharmacol. 70, 1405–1412. 10.1007/s00228-014-1747-1 25228250

[B10] Finnish Medicines Agency (2022c). FimeaWeb. Available at: https://www.fimea.fi/web/en/databases_and_registers/fimeaweb (Accessed April 12, 2022).

[B11] Finnish Medicines Agency (2022b). Shortages. Available at: https://www.fimea.fi/web/en/databases_and_registers/shortages (Accessed April 12, 2022).

[B12] Finnish Medicines Agency (2022a). Supervision. Available at: https://www.fimea.fi/web/en/supervision (Accessed April 12, 2022).

[B13] Finnish National Board on Research Integrity (2022). Ethical review in Finland . Available at: https://tenk.fi/en/ethical-review/ethical-review-finland (Accessed April 12, 2022).

[B14] Fox E. R. Tyler L. S. (2017). Potential association between drug shortages and high-cost medications. Pharmacotherapy 37, 36–42. 10.1002/phar.1861 27891635

[B15] Garattini L. Badinella Martini M. Mannucci P. M. (2022). Pharmaceutical patenting in the european union: reform or riddance. Intern Emerg. Med. 17, 937–939. 10.1007/s11739-021-02887-6 34783000 PMC8592279

[B16] Heiskanen K. Ahonen R. Kanerva R. Karttunen P. Timonen J. (2017). The reasons behind medicine shortages from the perspective of pharmaceutical companies and pharmaceutical wholesalers in Finland. PLoS One 12, e0179479. 10.1371/journal.pone.0179479 28658307 PMC5489167

[B17] Heiskanen K. Ahonen R. Karttunen P. Kanerva R. Timonen J. (2015). Medicine shortages--a study of community pharmacies in Finland. Health Policy 119, 232–238. 10.1016/j.healthpol.2014.11.001 25467285

[B18] Kruuti J. (2021). “Medicine reimbursement system and approval of medicine prices,” in Finnish Statistics on Medicines 2020 (Helsinki, Finland: The Social Insurance Institution of Finland and the Finnish Medicines Agency). Available at: https://www.julkari.fi/bitstream/handle/10024/143552/Finnish_statistics_on_medicines_2020.pdf?sequence=1andisAllowed=y (Accessed May 4, 2022).

[B19] Langedijk J. Whitehead C. J. Slijkerman D. S. Leufkens H. G. Schutjens M. H. Mantel-Teeuwisse A. K. (2016). Extensions of indication throughout the drug product lifecycle: a quantitative analysis. Drug Discov. Today 21, 348–355. 10.1016/j.drudis.2015.11.009 26657087

[B20] Mazer-Amirshani M. Pourmand A. Singer S. Pines J. M. van den Anker J. (2014). Critical drug shortages: implications for emergency medicine. Acad. Emerg. Med. 6, 704–711. 10.1111/acem.12389 25039558

[B21] Musazzi U. M. Di Giorgio D. Minghetti P. (2020). New regulatory strategies to manage medicines shortages in Europe. Int. J. Pharm. 579, 119171. 10.1016/j.ijpharm.2020.119171 32092455 PMC7125892

[B22] Närhi U. Asola E. (2021). “Pharmaceutical policies,” in Finnish Statistics on Medicines 2020 (Helsinki, Finland: The Social Insurance Institution of Finland and the Finnish Medicines Agency). Available at: https://www.julkari.fi/bitstream/handle/10024/143552/Finnish_statistics_on_medicines_2020.pdf?sequence=1andisAllowed=y (Accessed May 4, 2022).

[B23] Palva E. (2015). Lääketurvallisuuden varmistaminen – haittavaikutusseurantaa ja aktiivista ennakointia [in Finnish]. Available at: https://sic.fimea.fi/documents/721167/868092/29301_2_15_19-21_Laaketurvallisuuden_valmistaminen.pdf (Accessed April 12, 2022).

[B24] Pauwels K. Huys I. Casteels M. Simoens S. (2014). Drug shortages in european countries: a trade-off between market attractiveness and cost containment? BMC Health Serv. Res. 14, 438. 10.1186/1472-6963-14-438 25257912 PMC4263120

[B25] Phuong J. M. Penm J. Chaar B. Oldfield L. D. Moles R. (2019). The impacts of medication shortages on patient outcomes: A scoping review. PLoS One 14, e0215837. 10.1371/journal.pone.0215837 31050671 PMC6499468

[B26] Ruskoaho H. (2018). Development of the medicine reimbursement scheme. Examiner’s final report. Reports and memos of the ministry of social affairs and health 20/2018. Available at: https://julkaisut.valtioneuvosto.fi/bitstream/handle/10024/160908/STM_20_Laakekorvausjarjestelman_kehittaminen_WEB.pdf?sequence=1andisAllowed=y (Accessed April 21, 2022).

[B27] Shukar S. Zahoor F. Hayat K. Saeed A. Gillani A. H. Omer S. (2021). Drug shortage: causes, impact, and mitigation strategies. Front. Pharmacol. 12, 693426. 10.3389/fphar.2021.693426 34305603 PMC8299364

[B28] Stevens W. Incerti D. Peneva D. Shrestha A. Smith G. Ramaswamy K. (2020). An empirical investigation of time-varying cost-effectiveness across the product life cycle. Health Econ. 29, 580–590. 10.1002/hec.4004 32083778

[B29] Tapanila T. Kari H. Koskinen H. (2021). A replacement medicine is available in most drug shortage cases. Suom. Laakaril. 76, 1161–1165.

[B30] The Social Insurance Institution of Finland (2022a). Medicinal products database. Available at: https://asiointi.kela.fi/laakekys_app/LaakekysApplication?kieli=en (Accessed April 12, 2022).

[B31] The Social Insurance Institution of Finland (2022b). Operations. Available at: https://www.kela.fi/web/en/kelas-operations (Accessed April 12, 2022).

[B32] The Social Insurance Institution of Finland (2022c). Tilastotietokanta kelasto. Available at: https://www.kela.fi/kelasto (Accessed April 12, 2022).

[B33] U.S. Food and Drug Administration (2019). Drug shortages: root causes and potential solutions 2019. Available at: https://www.fda.gov/media/131130/download (Accessed April 12, 2022).

[B34] Videau M. Lebel D. Bussières J. F. (2019). Drug shortages in Canada: data for 2016-2017 and perspectives on the problem. Ann. Pharm. Fr. 77, 205–211. 10.1016/j.pharma.2018.11.007 30670298

[B35] World Health Organization (2022). Anatomic therapeutic chemical (ATC) classification. Available at: https://www.who.int/tools/atc-ddd-toolkit/atc-classification (Accessed April 12, 2022).

[B36] Zhang W. Guh D. P. Sun H. Lynd L. D. Hollis A. Grootendorst P. (2020). Factors associated with drug shortages in Canada: a retrospective cohort study. CMAJ Open 8, E535–E544. 10.9778/cmajo.20200036 PMC7641197 32873582

