# Supplementary material for: Medicine shortages: Product life cycle phases and characteristics of medicines in short supply—A register study
Source: Front Pharmacol. 2022 Jun 27;13:943249. doi: 10.3389/fphar.2022.943249 (PMC9272073; doi:10.3389/fphar.2022.943249)
Supplement: Supplementary file 1 [file Table1.docx]

Supplementary Table 1. The number of medicines shortages in Finland in 2017–2019 according to the Anatomic Therapeutic Chemical (ATC) category and 3-digit ATC class^1^ of medicines in short supply.

| ATC category and 3-digit class | Number of shortages, n (% of all reported shortages, N=3526) |
| --- | --- |
| **A**  A01  A02  A03  A04  A05  A06  A07  A09  A10  A11  A12  A14  A16 | **313 (8.9%)**  5 (0.1%)  89 (2.5%)  19 (0.5%)  13 (0.3%)  9 (0.3%)  29 (0.8%)  35 (1.0%)  7 (0.2%)  58 (1.6%)  17 (0.5%)  30 (0.9%)  1 (0.0%)  1 (0.0%) |
| **B**  B01  B02  B03  B05  B06 | **133 (3.8%)**  62 (1.8%)  18 (0.5%)  10 (0.3%)  42 (1.2%)  1 (0.0%) |
| **C**  C01  C02  C03  C05  C07  C08  C09  C10 | **698 (19.8%)**  29 (0.8%)  26 (0.7%)  22 (0.6%)  23 (0.7%)  63 (1.8%)  78 (2.2%)  342 (9.7%)  115 (4.4%) |
| **D**  D01  D03  D05  D06  D07  D08  D10  D11 | **185 (5.2%)**  18 (0.5%)  8 (0.2%)  13 (0.3%)  16 (0.5%)  66 (1.9%)  18 (0.5%)  23 (0.7%)  23 (0.7%) |
| **G**  G01  G02  G03  G04 | **325 (9.2%)**  17 (0.5%)  12 (0.3%)  162 (4.6%)  134 (3.8%) |
| **H**  H01  H02  H03  H05 | **64 (1.8%)**  22 (0.6%)  18 (0.5%)  1 (0.0%)  23 (0.7%) |
| **J**  J01  J02  J04  J05  J06  J07 | **195 (5.5%)**  116 (3.3%)  15 (0.4%)  1 (0.0%)  32 (0.9%)  7 (0.2%)  24 (0.7%) |
| **L**  L01  L02  L03  L04 | **219 (6.2%)**  116 (3.3%)  41 (1.2%)  10 (0.3%)  52 (1.5%) |
| **M**  M01  M02  M03  M04  M05  M09 | **139 (3.9%)**  83 (2.4%)  7 (0.2%)  19 (0.5%)  9 (0.3%)  18 (0.5%)  3 (0.1%) |
| **N**  N01  N02  N03  N04  N05  N06  N07 | **928 (26.3%)**  40 (1.1%)  287 (8.1%)  102 (2.9%)  36 (1.0%)  192 (5.4%)  239 (6.8%)  32 (0.9%) |
| **P**  P01  P02 | **7 (0.2%)**  3 (0.1%)  4 (0.1%) |
| **R**  R01  R02  R03  R05  R06 | **155 (4.4%)**  22 (0.6%)  6 (0.2%)  54 (1.5%)  11 (0.3%)  62 (1.8%) |
| **S**  S01  S02 | **90 (2.6%)**  85 (2.4%)  5 (0.1%) |
| **V**  V01  V03  V04  V07  V08  V09  V10 | **75 (2.1%)**  16 (0.5%)  17 (0.5%)  3 (0.1%)  12 (0.3%)  18 (0.5%)  8 (0.2%)  1 (0.0%) |

^1^ Reference for ATC classification: World Health Organization 2022
