# Supplementary material for: Medicine shortages: Product life cycle phases and characteristics of medicines in short supply—A register study
Source: Front Pharmacol. 2022 Jun 27;13:943249. doi: 10.3389/fphar.2022.943249 (PMC9272073; doi:10.3389/fphar.2022.943249)
Supplement: Supplementary file 2 [file Table2.DOCX]

Supplementary Table 2. Pairwise comparisons in the number of medicine shortages in different age groups. Statistically meaningful differences (p<0.05) between groups are marked with •.

| Age in years | 0-4 | 5-9 | 10-14 | 15-19 | 20-24 | 25-29 | 30-34 | 35-39 | 40-44 | 45-49 | 50-54 | 55 or more |
| --- | --- | --- | --- | --- | --- | --- | --- | --- | --- | --- | --- | --- |
| 0-4 |  | • | • | • | • | • | • | • | • | • | • | • |
| 5-9 | • |  | - | • | • | • | • | • | - | • | • | - |
| 10-14 | • | - |  | • | • | • | - | - | - | - | • | - |
| 15-19 | • | • | • |  | - | • | • | • | • | • | • | • |
| 20-24 | • | • | • | - |  | - | - | • | • | - | - | • |
| 25-29 | • | • | • | • | - |  | - | - | - | - | - | - |
| 30-34 | • | • | - | • | - | - |  | - | - | - | - | - |
| 35-39 | • | • | - | • | • | - | - |  | - | - | - | - |
| 40-44 | • | - | - | • | • | - | - | - |  | - | - | - |
| 45-49 | • | • | - | • | - | - | - | - | - |  | - | - |
| 50-54 | • | • | • | • | - | - | - | - | - | - |  | - |
| 55 or more | • | - | - | • | • | - | - | - | - | - | - |  |
