# Supplementary material for: Medicine shortages: Product life cycle phases and characteristics of medicines in short supply—A register study
Source: Front Pharmacol. 2022 Jun 27;13:943249. doi: 10.3389/fphar.2022.943249 (PMC9272073; doi:10.3389/fphar.2022.943249)
Supplement: Supplementary file 3 [file Table3.docx]

Supplementary Table 3. The number of medicine shortages in Finland in 2017–2019 (N=3,526) according to the age of the products and according to the Anatomic Therapeutic Chemical (ATC) category^1^.

| Age of products in years | The number of shortages, n | The number of shortages according to the ATC category, n | | | | | | | | | | | | | | | |
| --- | --- | --- | --- | --- | --- | --- | --- | --- | --- | --- | --- | --- | --- | --- | --- | --- | --- |
|  |  | A | B | C | D | G | H | J | L | M | N | P | | R | | S | V |
| 0-4 | 27 | 4 | - | 8 | 2 | - | - | 4 | 2 | - | 4 | 2 | 1 | | - | | - |
| 5-9 | 93 | 13 | 3 | 9 | 1 | 20 | - | 2 | 8 | 2 | 13 | 1 | 4 | | 12 | | 5 |
| 10-14 | 191 | 20 | 5 | 50 | 2 | 17 | 1 | 6 | 10 | 8 | 53 | - | 11 | | 4 | | 4 |
| 15-19 | 652 | 37 | 7 | 107 | 35 | 71 | 27 | 16 | 14 | 55 | 230 | - | 38 | | 14 | | 1 |
| 20-24 | 818 | 20 | 48 | 219 | 5 | 85 | 3 | 39 | 87 | 28 | 225 | - | 37 | | 6 | | 16 |
| 25-29 | 523 | 29 | 16 | 139 | 46 | 37 | 6 | 26 | 16 | 8 | 140 | 2 | 24 | | 21 | | 13 |
| 30-34 | 209 | 28 | 2 | 61 | 8 | 12 | - | 23 | 9 | 4 | 52 | - | - | | 6 | | 4 |
| 35-39 | 198 | 37 | 5 | 18 | 2 | 17 | - | 29 | 31 | - | 52 | - | 6 | | 1 | | - |
| 40-44 | 87 | 6 | - | 14 | 15 | 4 | 1 | 8 | 10 | 12 | 6 | - | 4 | | 5 | | 2 |
| 45-49 | 150 | 4 | 1 | 17 | 24 | 15 | 7 | 8 | 7 | 12 | 48 | - | 3 | | 4 | | - |
| 50-54 | 503 | 76 | 44 | 56 | 44 | 47 | 18 | 31 | 25 | 10 | 82 | - | 27 | | 13 | | 30 |
| 55 or more | 75 | 39 | 2 | - | 1 | - | 1 | 3 | - | - | 23 | 2 | - | | 4 | | - |

^1^ Reference for ATC classification: World Health Organization 2022
