# Supplementary material for: Medicine shortages: Product life cycle phases and characteristics of medicines in short supply—A register study
Source: Front Pharmacol. 2022 Jun 27;13:943249. doi: 10.3389/fphar.2022.943249 (PMC9272073; doi:10.3389/fphar.2022.943249)
Supplement: Supplementary file 4 [file Table4.docx]

Supplementary Table 4. The number of medicine shortages in Finland in 2017–2019 according to the Anatomic Therapeutic Chemical (ATC) classification^1^.

| ATC category | **Medicine shortages in Finland in 2017-2019, n** | | |
| --- | --- | --- | --- |
|  | All | Reimbursable^3^ | Non-reimbursable^3^ |
| All | 3 526 | 2 689 | 837 |
| A | 313 | 194 | 119 |
| B | 133 | 75 | 58 |
| C | 698 | 643 | 55 |
| D | 185 | 122 | 63 |
| G | 325 | 219 | 106 |
| H | 64 | 61 | 3 |
| J | 195 | 93 | 102 |
| L | 219 | 176 | 43 |
| M | 139 | 111 | 28 |
| N | 928 | 812 | 116 |
| P | 7 | 2 | 5 |
| R | 155 | 116 | 39 |
| S | 90 | 51 | 39 |
| V | 75 | 14 | 61 |

^1^ Reference for ATC classification: World Health Organization 2022

^2^ In this study, the term “reimbursable” is used for a medicine/ATC class in which at least one product is reimbursable, regardless of the reimbursement status of other products in the class.

^3^ The term “non-reimbursable” is used when none of the products in the class is reimbursable.
